# Sensorless variable impedance control of robot joints with 3K gear reducers based on hybrid dynamics model and meta-policy optimization

**DOI:** 10.3389/frobt.2026.1923729

**Published:** 2026-09-15

**Authors:** Jiyu Sun, Xiaoshan Gao, Liang Yan

**Affiliations:** 1 The School of Automation Science and Electrical Engineering, Beihang University, Beijing, China; 2 The Ningbo Institute of Technology, Beihang University, Ningbo, China; 3 Tianmushan Laboratory, Hangzhou, China

**Keywords:** 3K gear reducer, robot joint, variable impedance control, dynamic model, meta-policy optimization, momentum observer, proximal policy optimization, sensorless control

## Abstract

Achieving both high-precision trajectory tracking and compliant physical interaction in collaborative robot joints equipped with 3K planetary gear reducers remains challenging under torque-sensorless conditions. This paper proposes a physics-informed data-driven joint dynamics modeling method and an associated meta-policy-optimized adaptive variable impedance control scheme to address this limitation. First, a hybrid dynamics model combining lumped rigid-body dynamics with a Multilayer Perceptron (MLP) for residual loss estimation is established. By incorporating power-flow direction features, the network identifies and predicts the time-varying transmission efficiency and nonlinear friction losses of the 3K reducer across motoring and generating quadrants. Second, a model-based feedforward torque compensation scheme is designed, and a variable-gain generalized momentum observer is developed to achieve stable estimation of external interaction torques. Building upon this, a force-responsive variable-stiffness impedance control law is formulated, and a proximal policy optimization (PPO) algorithm is employed within a contextual reinforcement learning architecture to search for optimal high-level meta-parameters, enabling adaptive adjustment of impedance parameters. Multi-condition dynamic interaction simulations demonstrate that the proposed hybrid modeling approach achieves a 40% improvement in joint torque prediction accuracy compared to the conventional momentum disturbance observer (MDOB) baseline. Furthermore, the adaptive variable stiffness control law actively adjusts the joint stiffness in response to estimated external forces, satisfying the dual requirements of high-precision tracking in free space and high compliance in contact space.

## 1 Introduction

Collaborative robots (cobots), composed of modular joints and links, have attracted considerable attention owing to their lightweight design, inherent safety features, and capacity to facilitate physical human–robot interaction (pHRI) (Sadrfaridpour and Wang, 2018; Vicentini, 2020; Keshvarparast et al., 2024). In pHRI scenarios, robot compliance is of paramount importance (Schumacher et al., 2019). To achieve highly compliant and safe interaction while mitigating collision risks, force control (Petrea et al., 2021; Dehio et al., 2022) and impedance control (Zhang and Xia, 2019; Yang et al., 2022; Laubscher and Sawicki, 2021) have been widely adopted to modulate the dynamic relationship between the robot and its environment. As the actuation source of cobots, the accuracy of joint-level impedance control directly determines the overall interaction performance of the manipulator (Azocar and Rouse, 2022; Zheng et al., 2021; Calanca et al., 2016). However, due to the torsional flexibility introduced by mechanical transmissions, nonlinear friction effects, and both internal and external disturbances, achieving high-performance joint compliance control remains a persistent challenge.

In recent years, substantial research efforts have been devoted to enhancing joint compliance. Albu-Schäffer et al. (Albu-Schäffer et al., 2004; Ott et al., 2004; Albu-Schäffer et al., 2007) proposed a full-state feedback controller utilizing both torque and position feedback, and employed motor inertia shaping to reduce the influence of friction. To further compensate for nonlinear disturbances, approaches such as model-free friction observers (Kim et al., 2019), low-pass-filter-based friction compensation (Tien et al., 2008), and dual-disturbance-observer-based admittance control (Wan et al., 2023) have been successively proposed. In addition, considering mechanical losses in the transmission chain, several studies have established detailed dynamic models of harmonic drive gears to capture their nonlinear behavior (Wolf and Iskandar, 2018; Yan et al., 2022). However, these purely physics-based analytical models often struggle to accurately capture the time-varying dynamic losses that evolve with operating conditions such as load, speed, and temperature. Furthermore, harmonic and RV reducers, which are commonly employed in conventional cobots, suffer from poor bidirectional transmission efficiency (Lopez Garcia et al., 2020; Zhang et al., 2023); when back-driven by external contact forces, they are prone to impedance behavior degradation, making them ill-suited for applications demanding high-transparency interaction. Consequently, 3K planetary gear transmissions, which combine a high reduction ratio with superior bidirectional efficiency, have emerged as a promising hardware alternative (Du et al., 2024).

For the control of 3K gear-driven joints, existing works have made progress but still exhibit limitations. (Dai et al., 2025) proposed an adaptive robust torque control (ARTC) scheme based on multiple torque sensors, which achieves high-precision torque tracking but at the cost of redundant physical sensors in series. (Liu et al., 2025) developed an efficiency-based accurate modeling method; however, the computation of equivalent efficiency still relies on real-time torque sensor feedback, thus failing to realize fully sensorless operation. (Zhang et al., 2024) proposed a dynamic model considering forward and backward transmission efficiencies to achieve compliant control of 3K joints, but their approach adopts a steady-state equilibrium assumption and neglects the inertial torque on the link side, precluding real-time observation of external torques and rendering the compliant performance inadequate for complex dynamic interaction tasks. Moreover, the aforementioned analytical models generally struggle to accurately capture the load-dependent nonlinear losses, and they lack a variable impedance adaptation mechanism for unknown physical interaction tasks.

To address the above limitations, this paper proposes a torque-sensorless adaptive variable impedance control scheme designed for 3K gear-driven robot joints. The main contributions of this work are as follows:

- Hybrid dynamics modeling combining physics priors and a multilayer perceptron (MLP) for residual loss estimation: A hybrid modeling framework is proposed to address the nonlinear friction and time-varying transmission efficiency of 3K reducers that are difficult to capture analytically. The framework employs a lumped rigid-body model to preserve the fundamental inertial dynamics, while a multilayer perceptron implicitly approximates the nonlinear dissipation. This approach effectively circumvents complex parameter identification procedures and significantly improves joint torque estimation accuracy under the sensorless configuration.
- Hybrid model-based feedforward compensation and generalized momentum observer (GMOB) for external force estimation: Feedforward torque compensation is introduced to reduce the disturbance rejection burden of the feedback controller. Meanwhile, by replacing the high-frequency-noise-sensitive angular acceleration computation with integration of the system momentum, accurate and stable estimation of external interaction torques is achieved.
- Adaptive variable impedance control strategy based on meta-policy optimization: To overcome the limitation that fixed impedance parameters cannot simultaneously satisfy trajectory tracking precision and compliant interaction performance, a variable stiffness impedance control law based on the amplitude envelope of the estimated external torque is designed. The proximal policy optimization (PPO) algorithm is employed to search for optimal impedance meta-parameters within the physics-informed data-driven simulation environment, while the target damping term is dynamically coupled online, ensuring smoothness and stability during interaction.

The remainder of this paper is organized as follows. Section 2 establishes the hybrid joint dynamics model based on physics priors and residual compensation. Section 3 details the controller design, including model-based feedforward torque compensation, momentum observer for external torque estimation, and the meta-policy-optimized variable impedance control scheme. Section 4 validates the effectiveness of the proposed control scheme through simulation studies. Section 5 concludes the paper.

## 2 Driven joint dynamics modeling

For a joint module equipped with a 3K planetary gear reducer, the complex internal gear meshing causes the transmission efficiency to exhibit pronounced nonlinear characteristics that vary significantly with load, speed, and temperature (Wolf and Iskandar, 2018; Lopez Garcia et al., 2020). The conventional double-inertia flexible joint model requires an additional link-side absolute encoder (Albu-Schäffer et al., 2007), which complicates the mechanical co-design and increases cost. To address the problem of high-precision torque observation under sensorless conditions, this paper proposes a hybrid modeling framework that combines lumped dynamics with multilayer perceptron. The mechanical structure of a single joint and the joint drive system are illustrated in Figures 1, 2, respectively.

**FIGURE 1:**
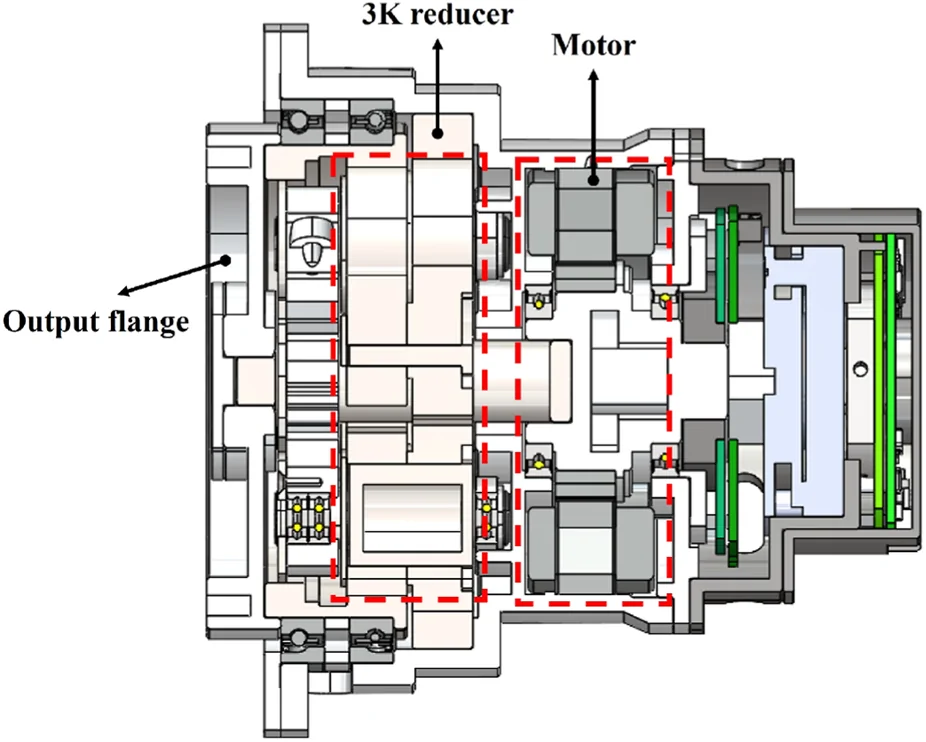
Mechanical structure of the single joint (Zhang et al., 2024).

**FIGURE 2:**
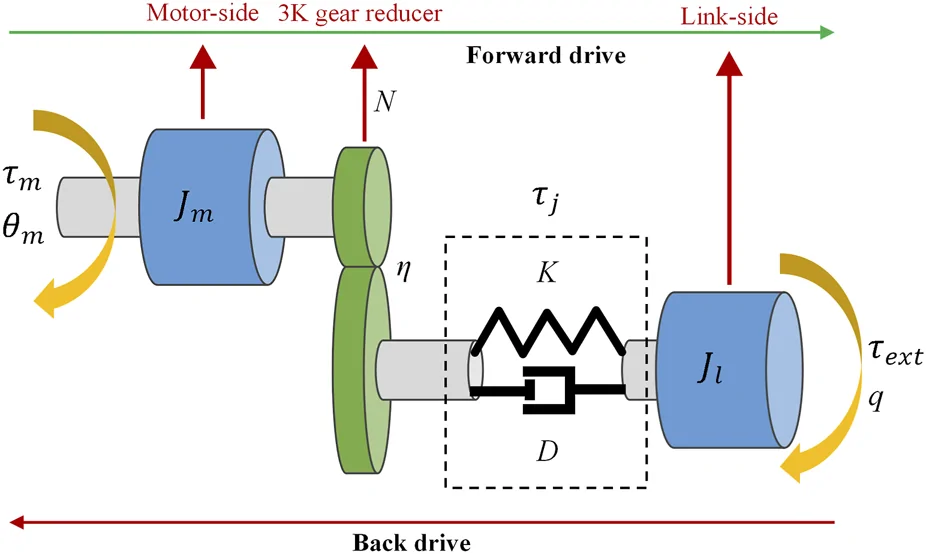
Diagram of the joint drive system.

Assuming the motor rotor and the link are rigidly connected and neglecting minor elastic deformations, the lumped dynamic equation on the joint side can be expressed as Equation 1:

$$J_{\text{nom}}\ddot{q}+\tau_{\text{fric}}\left(\dot{q}\right)+\tau_{\text{ext}}=\tau_{m},$$ (1)

where $J_{\text{nom}}$ denotes the lumped nominal inertia of the motor and link reflected to the joint side; $\ddot{q}$ is the joint angular acceleration; $\tau_{m}$ is the motor torque; $\tau_{\text{ext}}$ is the external interaction torque; and $\tau_{\text{fric}}\left(\dot{q}\right)$ represents the nonlinear frictional resistance within the system.

In actual physical transmission, the torque command issued by the servo cannot be fully converted into effective torque for driving the link or resisting external forces. There exist nonlinear losses caused by reducer hysteresis, nonlinear friction, and four-quadrant variable efficiency. In this work, these losses are collectively defined as the lumped torque loss $\tau_{\text{loss}}$ and are fitted using the multilayer perceptron. The Multi-Layer Perceptron network structure is illustrated in Figure 3. The true torque balance of the system is given by Equation 2:

$$J_{\text{nom}}\ddot{q}=\tau_{\text{cmd}}-\tau_{\text{loss}}-\tau_{\text{ext}},$$ (2)

where $\tau_{\text{cmd}}$ is the control torque command applied to the servo drive, and the lumped torque loss term is predicted by the multilayer perceptron $M_{\psi }$ as Equation 3:

$$\tau_{\text{loss}}=M_{\psi }\;\left(\dot{q},\;\tau_{\text{load}},\;\text{sign }\left(\tau_{\text{load}}\cdot \dot{q}\right)\right),$$ (3)

where $M_{\psi }$ is a multilayer perceptron. The inputs include the joint velocity $\dot{q}$ , the estimated load torque $\tau_{\text{load}}$ , and the power-flow direction $\text{sign }\left(\tau_{\text{load}}\cdot \dot{q}\right)$ . The network adopts a conventional fully connected feedforward architecture (3→64→32→1) with the hyperbolic tangent (tanh) activation function, ensuring first-order continuity of the torque compensation gradient in the control space.

**FIGURE 3:**
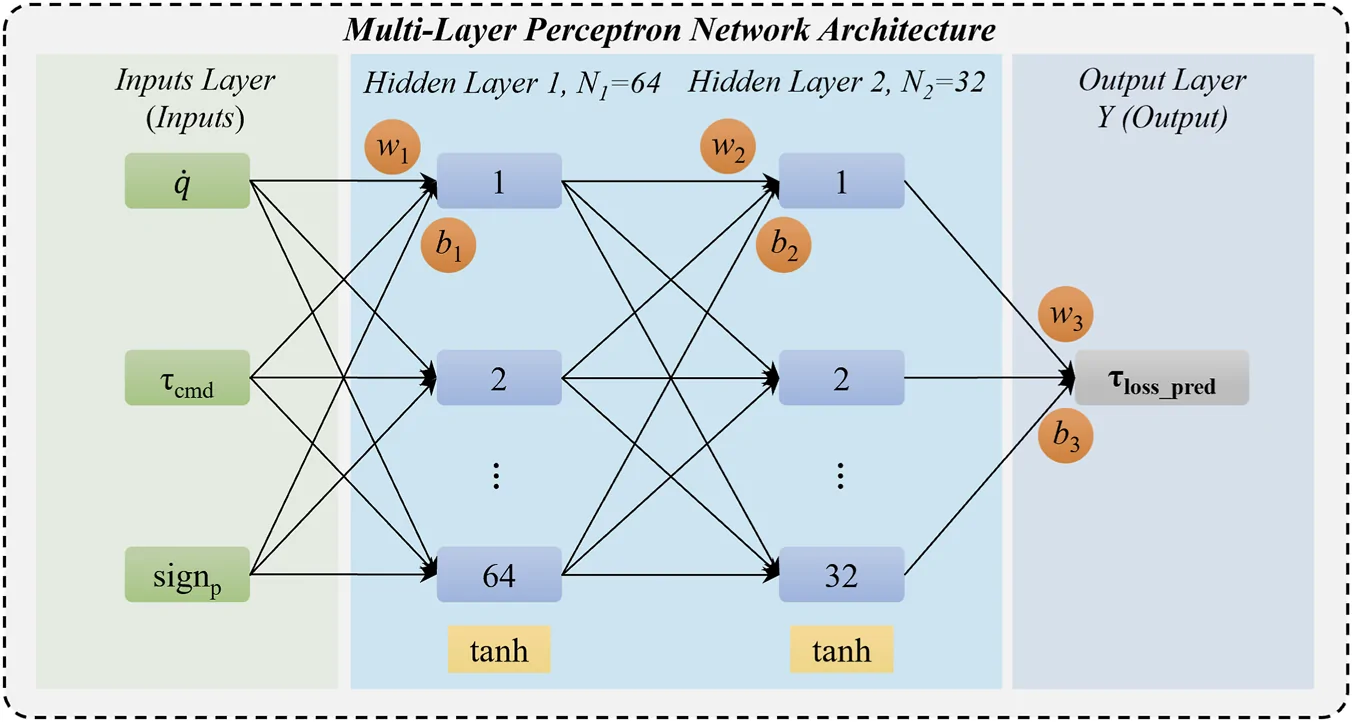
Multi-Layer Perceptron (MLP) network structure.

A key improvement lies in the design of the input feature vector $x={\left[\;\dot{q},\;\tau_{\text{cmd}},\;{\text{sign }}_{p}\;\right]}^{T}$ , in particular the introduction of a power-flow-based continuous feature operator ${\text{sign }}_{p}=\tanh\;\left(\frac{\tau_{\text{cmd}}\cdot \dot{q}}{\varepsilon }\right)$ (with ε=0.05 as the boundary layer smoothing parameter). As a physics-informed feature engineering approach, this operator exploits the physical significance of mechanical power polarity, enabling the network to identify and decouple the motoring and generating operating regimes of the system. This reduces network parameter overhead and improves cross-quadrant fitting efficiency, thereby achieving effective prediction for the asymmetric transmission characteristics of the 3K reducer.

During the offline training phase, to simulate high-fidelity complex joint characteristics, Stribeck friction and a four-quadrant efficiency model are explicitly employed in the underlying physics environment to generate ground-truth loss values for training the network. The network is supervised using the true lumped loss torque collected under time-series operating conditions, as defined in Equation 4:

$$\tau_{\text{loss\_true}}=\left(\tau_{\text{cmd}}-\tau_{\text{eff}}\right)+\tau_{\text{fric}},$$ (4)

where $\tau_{\text{fric}}$ is the Stribeck dynamic friction torque, and $\tau_{\text{eff}}$ is the theoretical effective electromagnetic torque calculated in the offline simulation environment based on the transmission efficiency model of the 3K reducer; in physical experiments, these offline dynamic loss terms can be directly substituted by the joint torque $\tau_{\text{sensor}}$ measured by a torque sensor.

Parameter optimization is performed based on the mean squared error (MSE) loss function in Equation 5:

$$L\left(\psi \right)=\frac{1}{N}\overset{N}{\underset{k=1}{\sum }}{\left(M_{\psi }\left(x_{k}\right)-\tau_{\text{loss\_true},k}\right)}^{2}.$$ (5)

To enhance the regressor’s generalization against thermal drifts and lubrication degradation, the training dataset is synthetically generated under a domain randomization (DR) scheme. Specifically, 100 frequency-swept excitation trajectories ( 0.1∼1.5 Hz for velocity and torque commands) are executed at a sampling rate of 1000 Hz while uniformly randomizing key joint parameters—including Coulomb/static friction $\left(F_{c},F_{s}\right)$ , viscous damping $\left(\sigma_{2}\right)$ , Stribeck velocity $\left(v_{s}\right)$ , and four-quadrant efficiencies $\left(\eta_{\text{nom}}\right)$ —within their bounded operational domains. This process yields 500,000 continuous time-series samples, which are subsequently split into an 80% training set and a 20% validation set. Regarding network architecture, hyperbolic tangent (tanh) activation functions are employed in the hidden layers instead of SiLU or ReLU to provide inherent mathematical saturation against anomalous transient impacts and to ensure a bias-free, odd-symmetric approximation of Coulomb friction reversals. The training converges stably across five independent random seeds (42, 123, 456, 789, 1024), and all hyperparameter configurations for the MLP regressor are summarized in Table 1.

**TABLE 1:** MLP training hyperparameter configuration.

MLP training hyperparameter/Item	Value/Description
Optimizer and Learning Rate	Adam, $\text{lr}=1\times 10^{-3}$ with exponential decay
Batch Size and Epochs	1024, 1200 epochs
Activation Function	Hyperbolic tangent (tanh)

Once the offline training phase is completed, the network parameters are frozen, and the model is deployed directly to the low-level joint servo control loop for online real-time feedforward compensation and joint torque estimation, operating without any gradient backpropagation or online parameter refinement within the control loop.

## 3 Controller design

### 3.1 Model-based feedforward torque compensation

The core objective of feedforward control is to compute the theoretical torque that the motor should provide, based on the desired trajectory, to counteract the system’s intrinsic dynamics and external disturbances, thereby achieving high-precision trajectory tracking. The feedforward torque is decomposed into a rigid-body dynamics feedforward term based on the analytical nominal dynamic equations, and a residual compensation feedforward term based on the MLP-approximated nonlinear friction and efficiency losses.

In the ideal feedforward control law design, it is assumed that the actual trajectory equals the desired trajectory. Feedforward primarily handles the self-dynamics, and unknown external interaction torques are temporarily not considered (external forces are handled by the feedback term). Under this assumption, the nominal rigid-body dynamics feedforward torque is defined as Equation 6:

$$\tau_{\text{rb}}=J_{\text{nom}}\;{\ddot{q}}_{d}.$$ (6)

Furthermore, to eliminate the losses arising from nonlinear friction and time-varying transmission efficiency of the 3K reducer, the offline-trained MLP is utilized to approximate the residual compensation feedforward torque. Since the equivalent internal load on the joint side cannot be directly measured during online operation, this paper adopts the sum of the nominal rigid-body feedforward torque and the external torque estimated by the momentum observer as an approximation of the joint internal load in Equation 7:

$${\tilde{\tau }}_{\text{load}}=\tau_{\text{rb}}+\tau_{\text{obs}}.$$ (7)

Thus, the residual compensation feedforward torque is expressed as Equation 8:

$$\tau_{\text{loss}}^{\text{pred}}=M_{\psi }\;\left({\dot{q}}_{d}\left(t\right),\;{\tilde{\tau }}_{\text{load}}\left(t\right),\;\text{sign }\;\left({\tilde{\tau }}_{\text{load}}\left(t\right)\cdot {\dot{q}}_{d}\left(t\right)\right)\right),$$ (8)

where ${\dot{q}}_{d}$ is the desired velocity; ${\tilde{\tau }}_{\text{load}}$ is the approximate load torque; and $\text{sign }\left({\tilde{\tau }}_{\text{load}}\left(t\right)\cdot {\dot{q}}_{d}\left(t\right)\right)$ is the power-flow direction.

The resultant feedforward torque is calculated by Equation 9:

$$\tau_{\text{ff}}=\tau_{\text{rb}}+\tau_{\text{loss}}^{\text{pred}}.$$ (9)

### 3.2 Variable-gain momentum observer for external force estimation

To realize external torque perception under sensorless conditions that rely solely on motor-side state feedback, this paper constructs an adaptive variable-gain generalized momentum observer (Ruder et al., 2014; De Luca et al., 2006). By integrating momentum, the observer avoids the computation of angular acceleration terms that are sensitive to high-frequency noise. The nominal generalized momentum of the joint is defined as Equation 10:

$$p=J_{\text{nom}}\;\dot{q}.$$ (10)

Differentiating the momentum with respect to time and substituting the dynamic equation yields (Equation 11):

$$\dot{p}=J_{\text{nom}}\;\ddot{q}=\tau_{\text{cmd}}-\tau_{\text{loss}}-\tau_{\text{ext}}.$$ (11)

By substituting the MLP-approximated lumped loss term, the effective joint driving torque is expressed as Equation 12:

$$\tau_{j}=\tau_{\text{cmd}}-\tau_{\text{loss\_pred}}.$$ (12)

The dynamic equation for the estimated external interaction torque r(t) (the observer output) is defined as Equation 13:

$$r\left(t\right)=k_{\text{obs}}\left(t\right)\left[\;p\left(t\right)-\int_{0}^{t}\left(\tau_{j}\left(s\right)+r\left(s\right)\right)\;ds-p\left(0\right)\right],$$ (13)

where $k_{\text{obs}}\left(t\right)$ is the time-varying observer gain, which determines the convergence rate of the observer, p(0) is the initial momentum, and $r\left(t\right)={\hat{\tau }}_{\text{ext}}\left(t\right)$ represents the estimated external interaction torque.

Taking the derivative of both sides and substituting the momentum derivative equation yields (Equation 14):

$$\dot{r}\left(t\right)=k_{\text{obs}}\left(t\right)\left(\dot{p}\left(t\right)-\tau_{j}\left(t\right)-r\left(t\right)\right)=k_{\text{obs}}\left(t\right)\left(\tau_{\text{ext}}\left(t\right)-r\left(t\right)\right).$$ (14)

Under the quasi-static assumption for transient interactions, the time-varying gain can be approximated as a constant baseline $K_{o}$ . Performing the Laplace transform on the equation yields (Equation 15):

$$R\left(s\right)=\frac{K_{o}}{s+K_{o}}\;T_{\text{ext}}\left(s\right).$$ (15)

This indicates that the output of the momentum observer is essentially the external torque filtered through a first-order low-pass filter.

To maintain a low observation bandwidth during quasi-static operation for filtering background noise, while widening the observation bandwidth during dynamic interactions to improve the response speed of external force tracking and achieve dynamically stable estimation of diverse external interaction torques, a variable-gain adaptive law is designed as Equation 16:

$$k_{\text{obs}}\left(t\right)=\left\{\begin{array}{cc} K_{\text{BASE}}, & |{\dot{\tau }}_{\text{filtered}}|\leq I_{\text{THRESH}}\\ \min\;\left(K_{\text{BASE}}+\Lambda_{\text{GAIN}}\cdot |{\dot{\tau }}_{\text{filtered}}|,\;K_{\text{MAX\_OBS}}\right), & |{\dot{\tau }}_{\text{filtered}}|>I_{\text{THRESH}} \end{array}\right.,$$ (16)

where $K_{\text{BASE}}=20$ (approx. 3.18 Hz ) is the baseline low-frequency gain to cover the dominant frequency band of physical human–robot interactions (typically below 2 Hz ) while attenuating the high-frequency quantization noise induced by encoder numerical differentiation; $\Lambda_{\text{GAIN}}=1.2$ is the adaptive amplification factor; and $K_{\text{MAX\_OBS}}=200$ (approx. 31.8 Hz ) is matched with the transient spectrum of rigid collisions (typically within 10∼30 Hz ), ensuring sufficient dynamic bandwidth to capture impact torques. The input variable ${\dot{\tau }}_{\text{filtered}}$ is the rate-of-change obtained after differencing the estimated external interaction torque, applying dead-zone suppression, and passing through a first-order low-pass filter; $I_{\text{THRESH}}=40$ Nm/s is the threshold constant that distinguishes steady-state tracking from external force interaction.

### 3.3 Adaptive variable impedance control based on meta-policy optimization

After establishing a hybrid dynamics model of the joint, a simulation environment is obtained that can accurately predict the system response under control torque commands and external interaction torques. Based on this environment, this section proposes a meta-policy optimization method based on reinforcement learning (PPO), aimed at resolving the conflict in which fixed impedance parameters cannot simultaneously satisfy “high-precision tracking in free space” and “high compliance in contact space.”

The impedance model is defined as Equation 17:

$$M_{d}\;\ddot{e}+B_{d}\left(t\right)\;\dot{e}+K_{p}\left(t\right)\;e=\tau_{\text{obs}}\left(t\right),$$ (17)

where $e=q_{d}-q$ is the position tracking error, $M_{d}$ is the desired inertia, $K_{p}\left(t\right)$ denotes the time-varying joint stiffness, $B_{d}\left(t\right)$ represents the dynamically coupled damping coefficient, and $\tau_{\text{obs}}\left(t\right)$ is the observed external interaction torque.

To realize the characteristic of “high stiffness in the absence of external forces, high compliance in the presence of external forces,” a variable stiffness control law based on external force perception is designed. To ensure continuity and smoothness of the control parameters, a variant of the sigmoid function is introduced as the stiffness modulation curve. This curve is uniquely determined by a set of meta-parameters in Equation 18:

$$K_{p}\left(t;\phi \right)=K_{\text{min}}+\frac{K_{\text{max}}-K_{\text{min}}}{1+\exp\;\left(\alpha \cdot \left(|\tau_{\text{obs}}|-\tau_{\text{thresh}}\right)\right)},$$ (18)

where the meta-parameter vector is $\phi =\left[\;\alpha ,\;\tau_{\text{thresh}}\;\right]$ ; α is the sensitivity of stiffness variation, determining the steepness of the transition from rigid to compliant; $\tau_{\text{thresh}}$ is the torque threshold that triggers compliant behavior; $K_{\text{max}}$ is the maximum stiffness when no external force is present; $K_{\text{min}}$ is the minimum stiffness for safety assurance.

In the control loop, the raw external force estimate output by the momentum observer is highly susceptible to contact zero-crossing effects, producing high-frequency jitter. If directly used for stiffness adaptation, it would trigger system chattering. Therefore, an asymmetric amplitude envelope detector E(⋅) is cascaded within the stiffness adaptation law. Let the discrete time step be $t_{k}$ , the input signal be the absolute value of the observer torque $x_{k}=|\tau_{\text{obs}}\left(t_{k}\right)|$ , and the detector output is defined as Equation 19:

$$E\left(\left|\tau_{\text{obs}}\left(t_{k}\right)\right|\right)=\max\;\left(x_{k},\;\gamma \cdot E\left(\left|\tau_{\text{obs}}\left(t_{k-1}\right)\right|\right)\right),$$ (19)

where the decay coefficient γ∈(0,1) . This operator ensures that the stiffness adaptation achieves a instantaneous yielding response to collisions, while maintaining a prescribed exponential decay envelope as the external torque diminishes.

To prevent underdamped oscillations or overdamped lag caused by drastic changes in stiffness $K_{p}$ , the damping parameter $B_{d}$ is not treated as an independent variable but is dynamically adjusted according to the critical damping ratio $\xi_{\text{opt}}$ as a function of stiffness in Equation 20:

$$B_{d}\left(t\right)=2\;\xi_{\text{opt}}\sqrt{\;M_{d}\;K_{p}\left(t;\phi \right)\;}.$$ (20)

The feedback component of the controller is designed to emulate a variable stiffness spring-damper system, with the feedback torque given by Equation 21:

$$\tau_{\text{fb}}=K_{p}\left(t\right)\;\left(q_{d}-q\right)+B_{d}\left(t\right)\;\left({\dot{q}}_{d}-\dot{q}\right).$$ (21)

The optimal meta-parameter search problem is formulated as a Markov decision process (MDP) and solved using a parameter-based policy optimization approach. Unlike conventional step-based reinforcement learning, which requires the agent to read instantaneous body states at millisecond-level frequencies, this method adopts a contextual policy search architecture to achieve frequency decoupling between high-level decision-making and low-level high-frequency control. The input state vector is no longer composed of instantaneous physical quantities; instead, the dynamic conditions of the entire task episode are encoded as a global context feature vector $s_{k}$ in Equation 22:

$$s_{k}=\left[\;C_{\text{traj}},\;C_{\text{dist}},\;C_{\text{amp}}\;\right],$$ (22)

where $C_{\text{traj}}$ is the desired trajectory profile features (e.g., stationary, sinusoidal, or complex trajectory); $C_{\text{dist}}$ is the external disturbance type feature (e.g., no disturbance, step collision, sinusoidal excitation); and $C_{\text{amp}}$ is the normalized amplitude feature of the anticipated disturbance.

Under this architecture, the agent observes the context feature $s_{k}$ at the initial time t=0 of each task episode and performs a single forward pass through the policy network to output a set of global impedance meta-parameters as the action. This action remains constant throughout the episode of duration $T_{\text{ep}}$ , as represented in Equation 23:

$$a_{k}=\phi_{k}=\left[\;\alpha ,\;\tau_{\text{thresh}}\;\right]\in R^{2}.$$ (23)

To ensure high-precision tracking in free space and to reduce the search space, the baseline maximum stiffness $K_{\text{max}}=1500$ Nm/rad and the critical damping ratio $\xi_{\text{opt}}=0.707$ are set as constant priors. The agent only performs dimensionality-reduced optimization over the compliance sensitivity features. The low-level variable impedance controller combines this fixed set of meta-parameters with the analytical function in (Wan et al., 2023) and the amplitude envelope detector to accomplish real-time stiffness adaptation in the high-frequency control loop.

The objective of the reinforcement learning agent is to optimize the meta-policy $\pi_{\theta }$ , which achieves an optimal balance between “high-precision trajectory tracking” and “compliant physical interaction” by understanding the context distribution across different task conditions. The instantaneous reward function $r_{t}$ at each time step t is designed as a piecewise function in Equation 24:

$$\begin{array}{cccc} & r_{t}=-w_{p1}\;{\left(q_{d}-q\right)}^{2}-w_{c1}\;\left(K_{\text{max}}-K_{p}\right),\; & & \left(|\tau_{\text{obs}}|<\tau_{\text{safe}}\right)\\ & r_{t}=-w_{p2}\;{\left(e_{\text{pos}}\right)}^{2}-w_{c2}\;\left(K_{p}-K_{\text{min}}\right)\;|\tau_{\text{obs}}|,\; & & \left(|\tau_{\text{obs}}|\geq \tau_{\text{safe}}\right) \end{array},$$ (24)

where $w_{p1}=30000$ and $w_{p2}=10$ are the position tracking error penalty weights in the free-space and contact regimes, respectively; $w_{c1}=0.02$ and $w_{c2}=0.05$ are the compliance deviation penalty weights; and $\tau_{\text{safe}}=1.0$ Nm is the safety threshold distinguishing free-space from contact interaction. In the free-space regime, a compliance deviation penalty is introduced to prevent unprovoked stiffness softening triggered by measurement noise; conversely, in the contact regime, a severe high-stiffness penalty is imposed to enforce the meta-policy to decrease joint stiffness for compliant shock mitigation. The total return for the entire episode is defined as $R=\sum_{t=0}^{T}r_{t}$ .

The proximal policy optimization (PPO) algorithm (Schulman et al., 2017) is employed to iteratively update the high-level meta-policy. Since this is a parameter-based optimization task, the forward propagation of the policy network (Actor) outputs the mean of the Gaussian distribution over meta-parameters, while the standard deviation of the distribution is treated as a state-independent trainable global parameter vector that participates in synchronous optimization. The clipped surrogate objective function is defined as Equation 25:

$$L^{\text{CLIP}}\left(\theta \right)=E_{t}\left[\;\min\;\left(r_{t}\left(\theta \right)\;{\hat{A}}_{t},\;\text{clip }\;\left(r_{t}\left(\theta \right),\;1-\varepsilon ,\;1+\varepsilon \right)\;{\hat{A}}_{t}\right)\right],$$ (25)

where $r_{t}\left(\theta \right)$ is the probability ratio between the new and old policies, ${\hat{A}}_{t}$ is the estimated advantage function, and ε=0.2 is the policy clipping coefficient. During the offline search for policy parameters, the overall optimization objective also incorporates the mean squared error loss of the value network (Critic) and a policy entropy regularization term to encourage environmental exploration. Through multiple rounds of mini-batch gradient descent, the optimal meta-parameter set $\phi^{*}$ that can generalize to diverse unknown external torque conditions is ultimately identified.

The complete set of hyperparameters and neural network configurations for the PPO meta-policy optimization is detailed in Table 2.

**TABLE 2:** PPO meta-policy optimization hyperparameter configuration.

Hyperparameter	Value/Setting
Network architecture (Actor and Critic)	2-layer MLP (64, 64) with ReLU
Optimizer and learning rate	Adam, $\text{lr}=3\times 10^{-4}$ (shared)
Batch size	16 episodes (48,000 steps per batch)
Update epochs per iteration	4
Clip coefficient (ϵ)	0.20
Gradient clipping ( $L_{2}$ max norm)	0.5
Loss weighting factors	$c_{v}=0.002$ (value), $c_{e}=0.01$ (entropy)
Advantage normalization	Yes (z-score)
Curriculum schedule	Linear ramp-up (first 60 iterations)
Total training iterations	200 (3,200 episodes total)

Finally, the control torque is composed of the hybrid dynamics feedforward term and the adaptive impedance feedback term, as shown in Equation 26:

$$\tau_{\text{cmd}}=\tau_{\text{ff}}+\tau_{\text{fb}}.$$ (26)

## 4 Simulation results

To clarify the physical configuration of the simulation environment, all dynamic evaluations in this section are performed on the joint drive system integrated with a link-side load inertia, directly corresponding to the mechanical schematic illustrated in Figure 2. Specifically, the mechanical plant is configured with a reflected motor rotor inertia at the joint side of $J_{m}=0.20\text{ kg}\cdot {\text{m}}^{2}$ and a link-side equivalent load inertia of $J_{l}=0.32\text{ kg}\cdot {\text{m}}^{2}$ , which together constitute a lumped nominal system inertia of $J_{\text{nom}}=J_{m}+J_{l}=0.52\text{ kg}\cdot {\text{m}}^{2}$ . This configuration provides a realistic operational condition for applying external interaction torques $\tau_{\text{ext}}$ and evaluating compliant behaviors, while avoiding the numerical integration divergence typically associated with an unladen motor shaft simulation.

### 4.1 Validation of hybrid dynamics modeling and MLP-based torque loss compensation

The joint torque estimation curves under the test trajectory are shown in Figure 4. It can be observed that the nominal rigid-body model (red dashed line) fails to track the abrupt changes in true torque (gray thick line) caused by variable efficiency and friction. In contrast, the hybrid dynamics model proposed in this paper (green thin line) exhibits high fidelity to the ground-truth profile.

**FIGURE 4:**
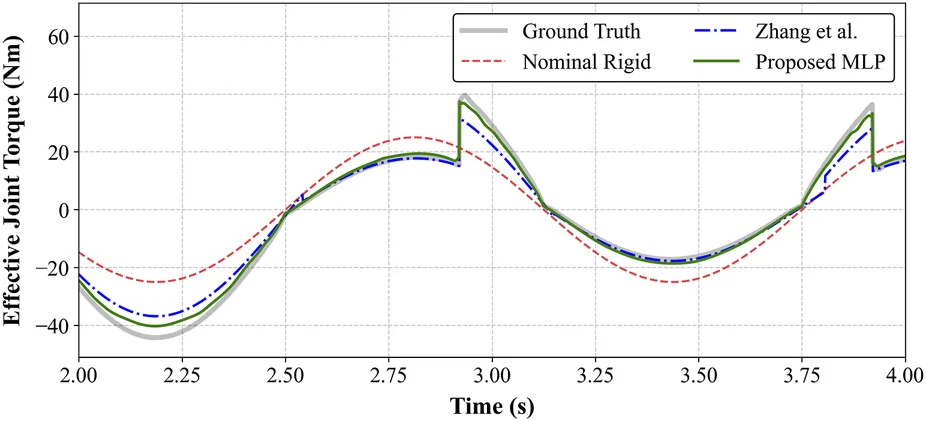
Validation of torque estimation.

To fairly and comprehensively evaluate the effectiveness of the proposed torque estimation method, a state-of-the-art approach proposed by (Zhang et al., 2024) is introduced as a comparative baseline. This baseline utilizes an analytical efficiency model combined with a momentum disturbance observer (MDOB) to estimate and compensate for joint losses. The statistical distributions of the Root Mean Square Error for torque estimation across multiple randomized scenarios, which include parameter drifts and hardware non-idealities, are presented in Figure 5 and summarized in Table 3.

**FIGURE 5:**
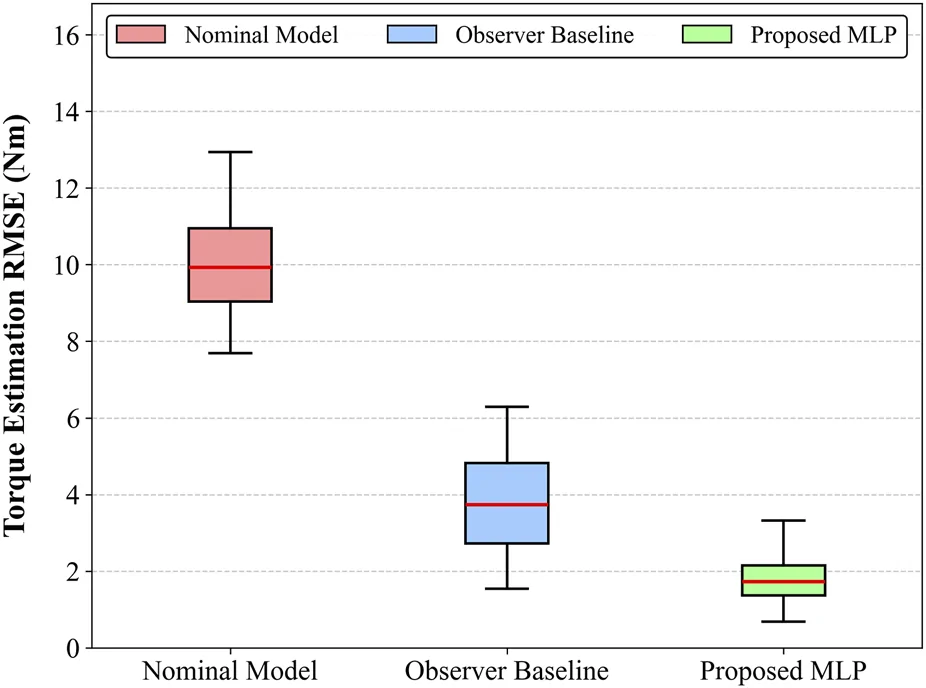
Statistical box-plot comparison of joint torque estimation RMSE across randomized scenarios.

**TABLE 3:** Statistical comparison of joint torque estimation RMSE across randomized scenarios.

Model	Torque prediction RMSE	Accuracy improvement
Nominal rigid-body model	9.9303 Nm	—
(Zhang et al., 2024) (MDOB-based)	3.7321 Nm	62.42%
Hybrid dynamics model (proposed)	1.7274 Nm	82.60%

The results indicate that the nominal rigid-body model, lacking loss compensation, exhibits a wide error distribution that is insufficient for contact tasks. The method by Zhang et al. effectively reduces the estimation error. However, limited by the phase lag inherent in the observer’s low-pass filter, its error distribution still shows noticeable fluctuations. In contrast, the proposed MLP hybrid model, functioning as a direct algebraic feedforward mapping, avoids observation delays. It demonstrates a lower error median and a narrower interquartile range (IQR) across diverse testing conditions, indicating improved estimation accuracy and robustness.

### 4.2 Validation of external torque observation and adaptive variable stiffness control

#### 4.2.1 Convergence analysis of meta-policy training

The average episodic return convergence curve during meta-policy training is shown in Figure 6. During the early training phase (first 30 episodes), as the agent is in the random exploration stage, unreasonable impedance configurations induce large tracking errors and safety penalties, causing a temporary decline in return values. As parameter optimization progresses, the policy network accurately establishes the control gradient, and the episodic return rapidly recovers around episode 60 and subsequently stabilizes, ultimately achieving steady convergence. This trend indicates that the high-level meta-policy has successfully learned the optimal adaptive control law, and can output reasonable impedance adaptation decisions in response to different external interaction characteristics.

**FIGURE 6:**
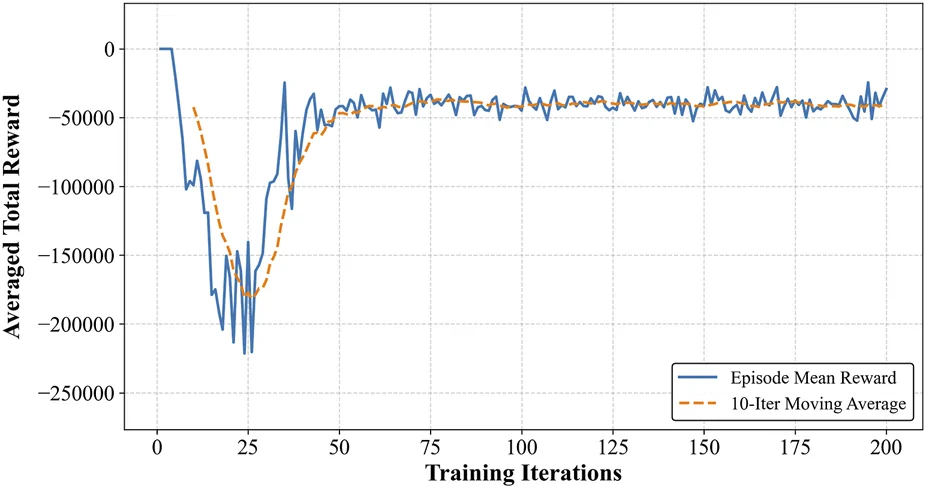
PPO meta-policy optimization convergence curve.

#### 4.2.2 Multi-condition dynamic interaction response analysis

To further verify the dynamic response performance of the momentum observer and the adaptive variable stiffness control law, this section conducts a comprehensive closed-loop simulation evaluation under two desired trajectory types—stationary (0–8 s) and sinusoidal motion (8–16 s)—each subjected to three typical external disturbance torques: sinusoidal, ramp, and step (applied during 2–4 s and 10–12 s, respectively). The system response curves are presented in Figures 7–12.

**FIGURE 7:**
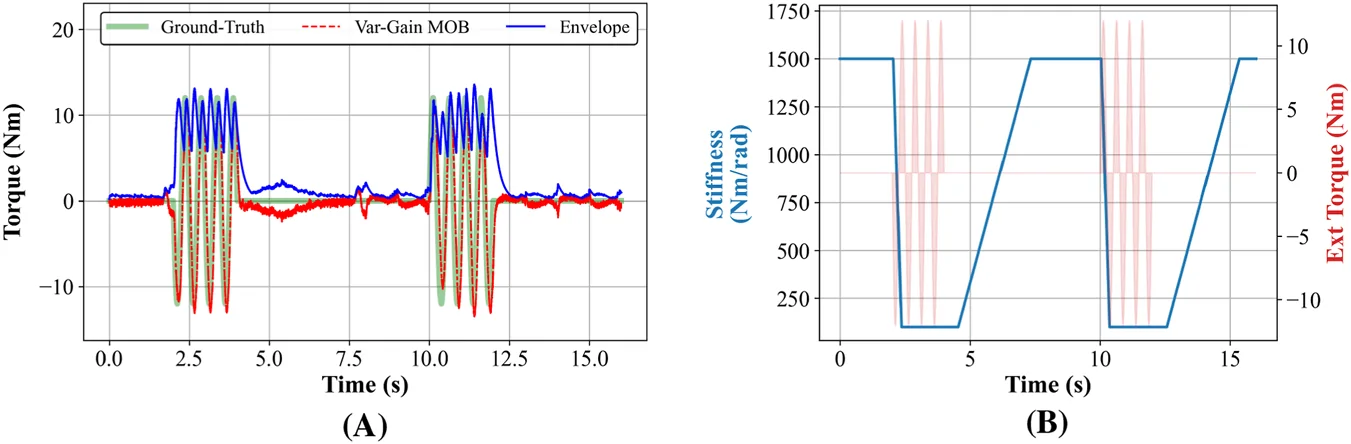
Estimation of external torque and response of variable stiffness control [Scenario: SINE]. **(A)** External torque estimation. **(B)** Variable stiffness response.

**FIGURE 8:**
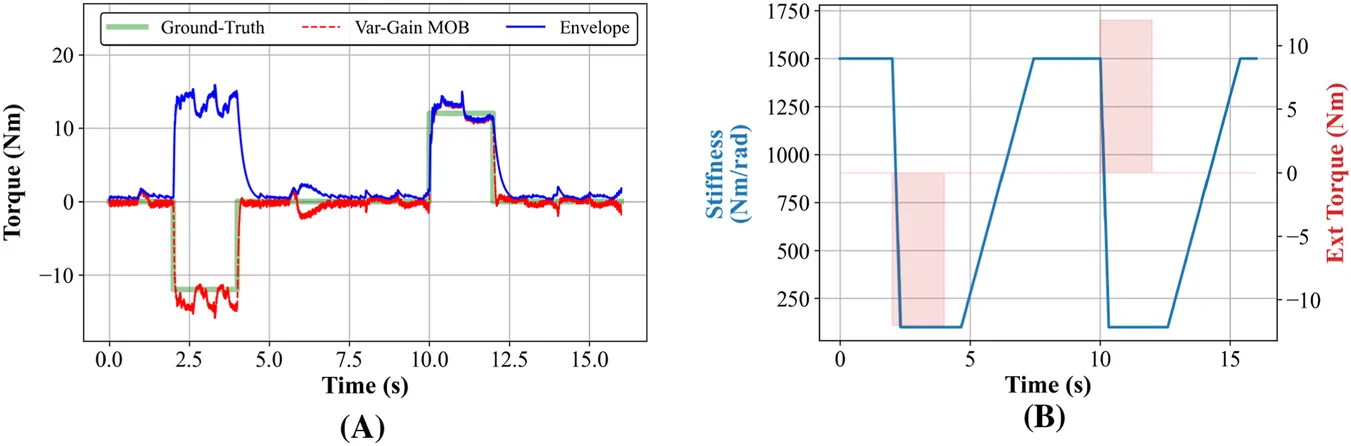
Estimation of external torque and response of variable stiffness control [Scenario: STEP]. **(A)** External torque estimation. **(B)** Variable stiffness response.

**FIGURE 9:**
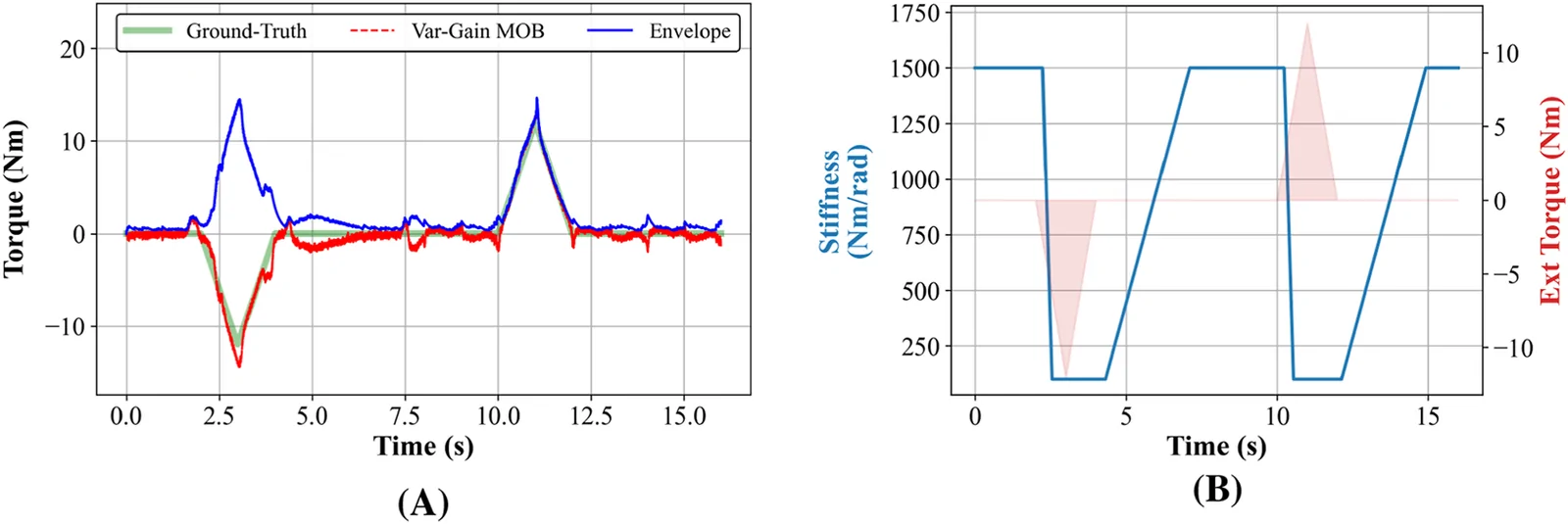
Estimation of external torque and response of variable stiffness control [Scenario: RAMP]. **(A)** External torque estimation. **(B)** Variable stiffness response.

**FIGURE 10:**
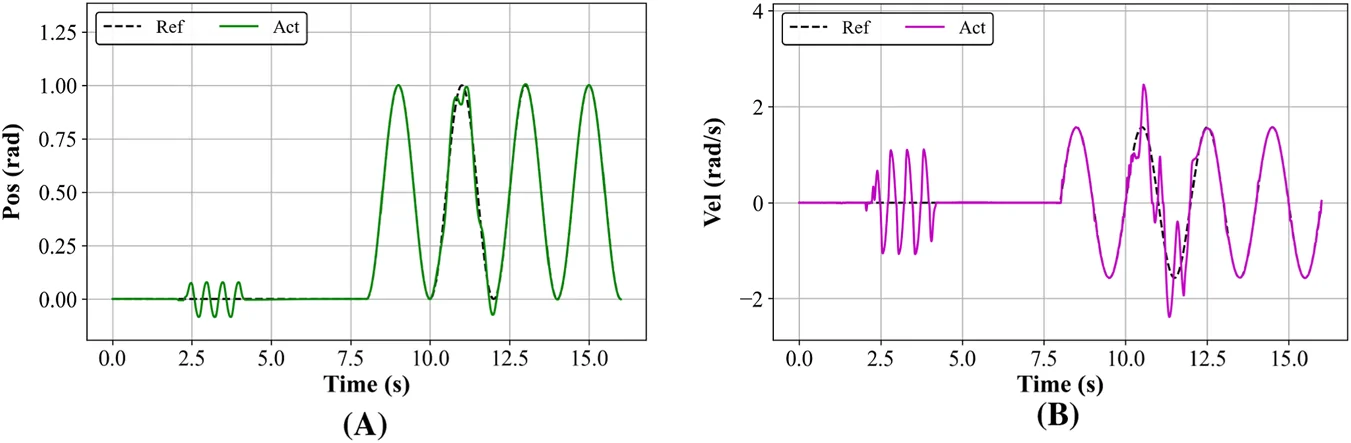
Trajectory tracking and compliant performances [Scenario: SINE]. **(A)** Position tracking. **(B)** Velocity tracking.

**FIGURE 11:**
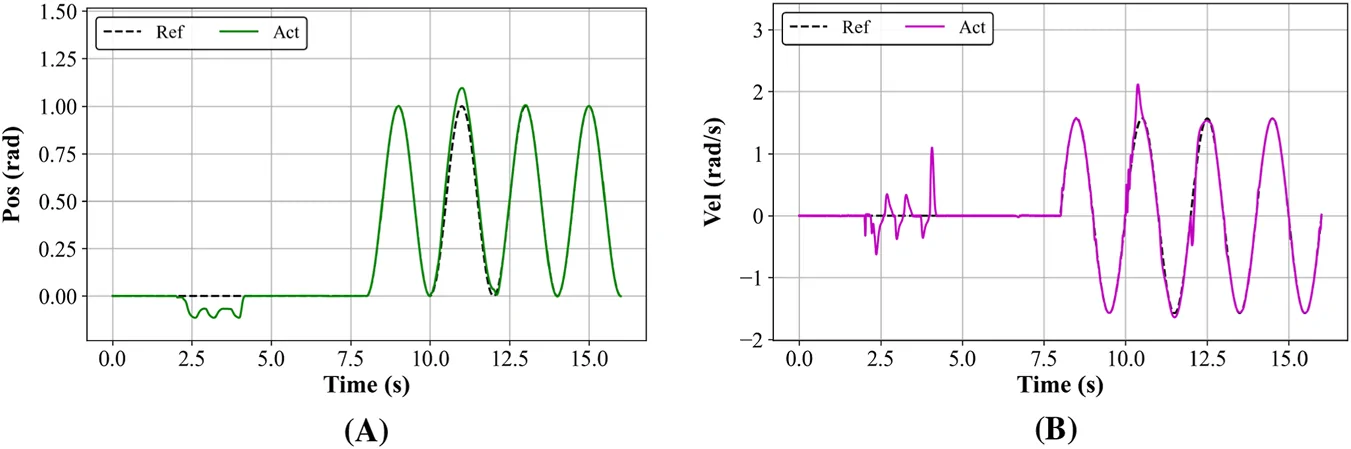
Trajectory tracking and compliant performances [Scenario: STEP]. **(A)** Position tracking. **(B)** Velocity tracking.

**FIGURE 12:**
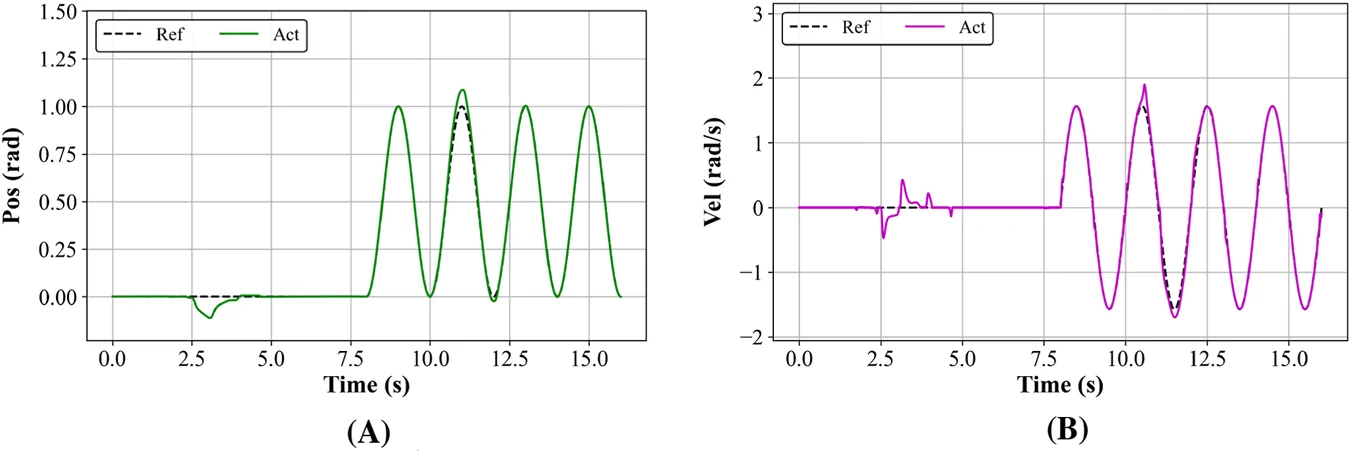
Trajectory tracking and compliant performances [Scenario: RAMP]. **(A)** Position tracking. **(B)** Velocity tracking.

As shown in Figures 7–9, although the injected hardware non-idealities (18-bit sensor quantization error, differentiation noise envelope, and 1–2 ms communication latencies) induce microscopic high-frequency fluctuations in the torque estimation, the observer accurately captures external interaction torques. By leveraging the combined protection of the observer deadband threshold and stiffness slew-rate constraints, low-level measurement noise is effectively isolated from the control loop. Consequently, the stiffness command stably maintains the 1500 Nm/rad baseline in free space; upon experiencing an external impact, it rapidly decreases to the 100 Nm/rad safety limit to absorb the collision before recovering smoothly at a linear rate once the disturbance is removed.

As shown in Figures 10–12, during disturbance-free intervals, the actual joint position and velocity smoothly track the reference trajectories, confirming the robustness of the MLP feedforward compensation against underlying hardware noise. During physical interaction intervals, active stiffness softening allows the joint to compliantly yield in the direction of the external force, effectively damping collision impacts. Once the external force is removed, the system recovers smoothly to precise trajectory tracking without overshoot, demonstrating the noise-rejection capability and practical engineering viability of the proposed framework in the presence of realistic hardware disturbances.

## 5 Conclusion

This paper proposed and validated a sensorless adaptive variable impedance control scheme for compliant joints driven by 3K planetary gear reducers. The research results indicate that combining a lumped rigid-body model with a multilayer perceptron (MLP) provides an effective approach to approximate the nonlinear friction and time-varying efficiency losses of the reducer, markedly improving joint torque estimation accuracy and supporting model-based feedforward torque compensation. Coupled with the variable-gain momentum observer, the simulation results demonstrate the feasibility of achieving dynamically stable estimation of external interaction torques using motor-side encoder feedback. Furthermore, the variable impedance control law, updated *via* the proximal policy optimization algorithm, can adjust the joint stiffness and damping in real time according to the characteristics of the interaction forces, allowing the system to avoid hard collision impacts from the environment while preserving dynamic tracking accuracy in free space.

This paper introduced methodologies to narrow the simulation-to-reality (Sim2Real) gap. During the offline training phase, a domain randomization (DR) strategy is introduced, enabling the MLP to learn robust, generalized nonlinear dissipation characteristics across uncertain physical boundaries. During the evaluation phase, a high-fidelity simulation environment explicitly incorporating hardware non-idealities is established, where the offline-trained frozen MLP loss model and the optimal PPO meta-parameters are deployed within the control loop, successfully validating the effectiveness of the proposed control algorithm. In future work, near-line transfer learning can be adopted to bridge the remaining Sim2Real gap, enabling real-world hardware adaptation.

## Data Availability

The raw data supporting the conclusions of this article will be made available by the authors, without undue reservation.
